# Impact of Personal Protective Equipment on Out-of-Hospital Cardiac Arrest Resuscitation in Coronavirus Pandemic

**DOI:** 10.3390/medicina57121291

**Published:** 2021-11-24

**Authors:** Hye-Young Ko, Jong-Eun Park, Da-Un Jeong, Tae-Gun Shin, Min-Seob Sim, Ik-Joon Jo, Gun-Tak Lee, Sung-Yeon Hwang

**Affiliations:** 1 Department of Emergency Medicine, Samsung Medical Center, Sungkyunkwan University School of Medicine, 81 Irwon-ro, Gangnam-gu, Seoul 06351, Korea; hyeyoung.ko@samsung.com (H.-Y.K.); jongeun7.park@samsung.com (J.-E.P.); daun.jeong@samsung.com (D.-U.J.); taegun.shin@samsung.com (T.-G.S.); minsub01.sim@samsung.com (M.-S.S.); ikjoon.jo@samsung.com (I.-J.J.); 2 Department of Emergency Medicine, College of Medicine, Kangwon National University, Chuncheon 24341, Korea

**Keywords:** powered air-purifying respirator, cardiac arrest, personal protective equipment

## Abstract

*BackgroundandObjectives*: This retrospective study evaluated the clinical impact of enhanced personal protective equipment (PPE) on the clinical outcomes in patients with out-of-hospital cardiac arrest. Moreover, by focusing on the use of a powered air-purifying respirator (PAPR), we investigated the medical personnel’s perceptions of wearing PAPR during cardiopulmonary resuscitation. *Materialsand Methods*: According to the arrival time at the emergency department, the patients were categorized into a conventional PPE group (1 August 2019 to 20 January 2020) and an enhanced PPE group (21 January 2020, to 31 August 2020). The primary outcomes of this analysis were the return of spontaneous circulation (ROSC) rate. Additionally, subjective perception of the medical staff regarding the effect of wearing enhanced PPE during cardiopulmonary resuscitation (CPR) was evaluated by conducting a survey. *Results*: This study included 130 out-of-hospital cardiac arrest (OHCA) patients, with 73 and 57 patients in the conventional and enhanced PPE groups, respectively. The median time intervals to first intubation and to report the first arterial blood gas analysis results were longer in the enhanced PPE group than in the conventional PPE group (3 min vs. 2 min; *p* = 0.020 and 8 min vs. 3 min; *p* < 0.001, respectively). However, there were no significant differences in the ROSC rate (odds ratio (OR) = 0.79, 95% confidence interval (CI): 0.38–1.67; *p* = 0.542) and 1 month survival (OR 0.38, 95% CI: 0.07–2.10; *p* = 0.266) between the two groups. In total, 67 emergent department (ED) professionals responded to the questionnaire. Although a significant number of respondents experienced inconveniences with PAPR use, they agreed that PAPR was necessary during the CPR procedure for protection and reduction of infection transmission. *Conclusion*: The use of enhanced PPE, including PAPR, affected the performance of CPR to some extent but did not alter patient outcomes. PAPR use during the resuscitation of OHCA patients might positively impact the psychological stability of the medical staff.

## 1. Introduction

Coronavirus disease (COVID-19), caused by severe acute respiratory syndrome coronavirus 2 (SARS-CoV-2), is an ongoing global public health threat with significant morbidity and mortality [1,2,3,4]. This pandemic poses considerable challenges, especially for healthcare professionals (HCPs) in the emergency department (ED) who are in the front lines to treat suspected or confirmed COVID-19 patients [5,6,7].

SARS-CoV-2 is a highly transmissible virus that spreads rapidly and primarily through droplets or even through aerosols in certain circumstances [8,9,10,11]. A recent meta-analysis showed that specific aerosol-generating procedures increase the risk of transmission of SARS-CoV-2 from patients to HCPs and that wearing appropriate personal protective equipment (PPE) reduces this risk [12]. Therefore, it is essential to wear enhanced PPE during the treatment of patients with suspected or confirmed COVID-19, especially when performing an aerosol-generating procedure [13].

It is not often possible to obtain sufficient information about a patient in case of out-of-hospital cardiac arrest (OHCA), making it difficult to determine whether the patient has COVID-19 [14,15,16]. Furthermore, it is common for patients with asymptomatic infections to transmit the virus to others [17,18,19]. Several procedures with the potential to generate aerosols, such as bag-mask ventilation, endotracheal intubation (ETI), suction, and chest compressions, are performed during cardiopulmonary resuscitation (CPR) [5,14,16]. Therefore, in the COVID-19 pandemic era, it is reasonable to wear enhanced PPE during the resuscitation of OHCA patients.

Several organizations have updated their guidelines for OHCA to cope with the COVID-19 pandemic, recommending the use of an appropriate level of PPE [14,15,20]. However, the cumbersome PPE could negatively affect the performance of the HCPs, both physically and psychologically. This is clinically relevant because hampering the ability of HCPs can adversely affect patient outcomes. There have been studies investigating the effects of wearing PPE during resuscitative procedures. However, most of them are simulation-based studies, and clinical studies are lacking. Furthermore, the results are mixed depending on the study design, population, and type of PPE [21,22,23,24,25]. In South Korea, the first case of COVID-19 was confirmed on 20 January 2020. In our institution, enhanced PPE was made mandatory for resuscitation of OHCA patients in the ED from 21 January 2020. This study aimed to evaluate the impact of the changes in clinical practice due to the COVID-19 outbreak, especially the use of enhanced PPE, on the outcomes of patients with OHCA. Additionally, we investigated the perceptions of the HCPs toward using enhanced PPE during resuscitation procedures.

## 2. Materials and Methods

### 2.1. Study Design and Setting

This was a single-center study conducted in the ED of Samsung Medical Center, which is a 1960-bed, university-affiliated, tertiary referral hospital with an annual census of 70,000 in Seoul, South Korea. This study comprised two sections. In the first section, we conducted a retrospective observational study to investigate the clinical impact of enhanced PPE on the tasks performed during CPR and the prognosis of the OHCA patients. According to the arrival time at the ED, the patients were categorized into a conventional PPE group (from 1 August 2019 to 20 January 2020) and an enhanced PPE group (from 21 January 2020 to 31 August 2020). The primary outcome of this analysis was the recovery of spontaneous circulation (ROSC) rate. In the second section, we conducted a survey regarding the perceptions of the HCPs towards the use of enhanced PPE.

### 2.2. Study Population

OHCA patients aged >18 years were included in this study. Patients who recovered spontaneous circulation at the pre-hospital stage were excluded. The HCPs who performed resuscitation procedures for OHCA patients in both groups were recruited for the survey.

### 2.3. Changes in the Level of PPE since the COVID-19 Pandemic

Before the COVID-19 pandemic, a surgical mask and rubber gloves with/without disposable, chlorinated polyethylene isolation gowns were used during CPR of OHCA patients. However, after the first COVID-19 patient was identified, HCPs participating in the CPR procedures for OHCA patients were obliged to wear enhanced PPE. This comprised a complete bodysuit or at least a waterproof surgical gown, apron, rubber gloves, boots, N95 respirators (inside the hood), and powered air-purifying respirator (PAPR). The PAPR used in this study consisted of a loose-fitting hood, breathing tube, high-efficiency particulate air filter, and blower. We used two types of PAPRs: 3M Jupiter Powered Air Turbo with a breathing tube (BT-20 L) and a loose-fitting hood (S-433 L-5) (3M, St. Paul, MN, USA) and an AIR WING III PAPR system with a hood kit (OTOS, Seoul, Korea).

### 2.4. Data Collection and Survey Development

We retrospectively reviewed the electronic medical records and our institutional registry for the OHCA patients’ data collected for CPR quality improvement. The following variables were retrieved: age, sex, preexisting conditions (cardiovascular disease, pulmonary disease, chronic kidney disease, malignancy, cerebrovascular accident (CVA), witnessed arrest, bystander CPR, arrest location, pre-hospital CPR duration, hospital CPR duration, initial electrocardiography (ECG) rhythm, intubation trial number, intubation success, time interval to first intubation trial, time interval to first arterial blood gas analysis (ABGA) achieved, targeted temperature management, ROSC, alive ED discharge, and 1 month survival.

Three investigators (JEP, GTL, and HYK) developed a questionnaire based on previously published literature and the perspectives of ED professionals. Two EM specialists (SYH and TGS) reviewed the questionnaire. The survey response options varied. Most of the questions related to CPR performance used the 5-point Likert scale for the responses. Multiple choices were used for questions related to the reason for the response, and opinions could be described freely in a separate section. The survey was provided to the participants as a paper document; no incentive was offered for participation.

### 2.5. Statistical Analysis

Data are represented as medians with interquartile ranges (IQR) for continuous variables and numbers and percentages for categorical variables. Wilcoxon rank-sum test or chi-squared test was performed, as appropriate. Multivariable logistic regression analysis was performed to evaluate the association between the use of enhanced PPE and clinical outcomes. The adjusted variables were selected based on clinical plausibility a priori and those exhibiting a significant difference between the conventional PPE group and enhanced PPE group. Preexisting CVA, witnessed or not, bystander CPR, arrest location, and initial ECG rhythm were included in the final model. The results are presented as odds ratios (OR) with 95% confidence intervals (CIs). Responses to the questionnaire using the 5-point Likert scale are presented as means ± standard deviations. A mean value ≥3 points indicated a significant trend. Responses to multiple-choice questionnaires are presented as numbers with percentages, and the results of the questionnaire using a numerical rating scale are represented by the average value. *p*-values < 0.05 were considered statistically significant. Data were analyzed using STATA software, version 15.1 (STATA Corporation, College Station, TX, USA).

## 3. Results

### 3.1. Impact of Enhanced PPE on CPR

A total of 130 OHCA patients were included in the study, of which whom 73 and 57 were classified into the conventional PPE and enhanced PPE groups, respectively. Baseline characteristics and outcome measurements of all patients are presented in Table 1.

The median age was 68 years (IQR, 57–81), and 56.1% (*n* = 75) were male. There were no significant differences in the sex or age between the two groups. Pre-hospital variables including witnessed arrest, bystander CPR, CPR duration, and arrest location did not differ significantly between the two groups. As compared to the conventional PPE group, the enhanced PPE group showed longer ED CPR duration (18 min (IQR, 12–23) vs. 15 min (IQR, 8–20); *p* = 0.030), longer time interval to first intubation (3 min (IQR 2–5) vs. 2 min (IQR 1–3); *p* = 0.020), and longer time interval to report the first ABGA results (8 min (IQR, 5–13) vs. 3 min (IQR, 1–8); *p* < 0.001). The first-pass success rate (90.0% vs. 78.4%; *p* = 0.130), rate of ROSC in the ED (49.3% vs. 43.8%; *p* = 0.597), survival until ED discharge (26.3% vs. 18.1%, *p* = 0.295), and 1 month survival (8.2% vs. 3.5%; *p* = 0.465) were all lower in the enhanced PPE group than in the conventional PPE group, although the difference was not statistically significant.

In multivariable logistic regression analyses, using enhanced PPE was not associated with the ROSC rate (OR = 0.79, 95% CI: 0.38–1.67; *p* = 0.542) or 1 month survival (OR = 0.38, 95% CI: 0.07–2.10; *p* = 0.266) (Table 2).

### 3.2. Survey

#### 3.2.1. Baseline Characteristics of Survey Respondents

In total, 67 HCPs in the ED responded to the survey. The baseline characteristics of the survey respondents are summarized in Supplementary Table S1. The average age was 31.7 years, and 45 (67.2%) were female. The distribution of the respondents according to the frequency of wearing PPE during CPR was as follows: <10 times, 54% (*n* = 36); 10–19 times, 34% (*n* = 23); and >20 times, 12% (*n* = 8).

#### 3.2.2. Perceptions of HCPs with Respect to Enhanced PPE Versus Conventional PPE

The survey results on wearing enhanced PPE during resuscitation of OHCA patients are summarized in Table 3 (see also Supplementary Table S2). In all, 70.1% of the HCPs responded that they either “agreed” or “strongly agreed” that the overall quality of CPR performance was negatively affected by enhanced PPE use. Specifically, the majority of respondents responded that certain procedures, including CPR instruction, ETI, chest compressions, and intravenous line insertion, were hampered with use of the enhanced PPE. On the other hand, they reported that drug administration, defibrillation, and patient monitoring were not affected much.

Overall, 82.1% and 68.7% of the respondents “strongly agreed” or “agreed” that the breathing was comfortable and that the heat buildup was appropriately suppressed while wearing enhanced PPE, respectively. However, 62.7% of the HCPs “strongly disagreed” or “disagreed” that it was easy to secure clear vision. The majority of the respondents reported difficulties in communication among themselves (94.0% responded with “strongly agreed” or “agreed”). They also reported that their movement was limited with enhanced PPE (79.1% responded with “strongly agreed” or “agreed”) and that it impeded each other’s movements (77.7% responded with “strongly agreed” or “agreed”). Only 1.5% and 4.5% of respondents, respectively, “strongly agreed” and “agreed” that donning and doffing enhanced PPE was easy.

Most of the respondents answered that enhanced PPE was necessary during resuscitation of OHCA patients (*n* = 61) for the following reasons (duplicate answers possible): (1) “I feel protected” (*n* = 27); (2) “There is a positive effect of reducing the spread of infection” (*n* = 24); and (3) “I do not know if it is more effective in blocking the transmission of infection than other protective equipment, but in the current situation, an excessive degree of protection is required” (*n* = 15) (see Supplementary Table S3). Furthermore, their free opinions on wearing enhanced PPE during resuscitation of OHCA patients are described in Supplementary Table S4.

## 4. Discussion

In this study, we found that wearing enhanced PPE, including PAPR, affected the resuscitation performance to some extent, including delays in reporting the first blood test results and in ETI; however, there was no statistically significant difference in the clinical prognosis represented by the ROSC rate and 1 month survival.

In previous studies, the ROSC rate or survival was lower during the pandemic than that before it [24,25,26]. During the pandemic period, less invasive airway management techniques, such as supraglottic airway and bag-valve-mask, were preferred [24,25,26]. Moreover, there were more initial non-shockable rhythms [24,25], but bystander CPR or advanced life support (ALS) implementations were fewer [26]. Unlike previous studies, in this study, there were no significant differences in the pre-hospital variables such as bystander witnessed, bystander CPR, and emergency medical services (EMS) resuscitation duration before and during the pandemic period.

In our study, the main change at the hospital level during the COVID-19 pandemic in the resuscitation of OHCA patients was wearing enhanced PPE. Cumbersome PPE inevitably hinders the performance of the HCPs. Several studies have shown that it also affects the performance of chest compression, intravenous cannulation, ETI, and defibrillation [27,28,29,30,31]. In addition to the deterioration of the performance of these procedures, various factors such as psychological factors affecting the HCPs and deterioration of communication ability can affect the patient’s prognosis. Despite the aforementioned factors, there were no differences in the clinical outcomes between the two groups in our study; there are several possible explanations for this. First, the enhanced PPE used in our institution might be relatively less cumbersome compared to the level “C” PPEs used in previous simulation studies. Particularly, hand dexterity is greatly affected by gloves. However, latex surgical gloves with a relatively limited effect on hand performance was used in this study. Second, our institution had previously encountered a large outbreak of the Middle East respiratory syndrome coronavirus infection a few years ago [32]. Since then, simulation trainings to prepare for infectious disease outbreak and education on wearing enhanced PPE have been conducted regularly; hence, wearing enhanced PPE was relatively familiar for the HCPs. Third, the performance of chest compressions can be greatly affected when wearing enhanced PPE. Deterioration of chest compression performance can greatly affect the patient’s prognosis. Our institution mainly utilized mechanical devices both before and during the pandemic; hence, the hindering effects of PPE may have been attenuated. Fourth, the effect of wearing enhanced PPE may have been underestimated, because most of the patients in this study had initial non-shockable rhythms with very poor prognosis. Finally, there was no time delay due to wearing PPE, because information of the OHCA patients arriving at the hospital was provided well in advance by EMS.

In a survey of HCPs with experience in resuscitation of OHCA patients while wearing PAPR, we found that though PAPR had advantages in heat tolerance, the HCPs faced significant challenges in several aspects. This included securing clear vision, risk of contamination, discomfort during donning and doffing, communication problems, limitation of mobility, and interference in movement amongst the staff. Our results support the findings of previous studies that PAPR has advantages in heat tolerance but disadvantages with respect to mobility and communication [33,34,35]. Additionally, the majority of the HCPs had difficulties with some detailed resuscitation procedures and concluded that wearing PAPR affected ALS performance. Interestingly, despite several disadvantages of PAPR, the majority of the HCPs agreed that it was necessary to wear PAPR in the resuscitation of OHCA patients during the COVID-19 pandemic. The main reasons for this were to reduce the possibility of infection transmission and to feel protected. This shows that the psychological burden of the medical staff regarding the fear of infection is significant.

This study has several limitations. First, this was a single-center and retrospective study conducted in the ED of a tertiary referral hospital. Thus, the results of this study may not be generalizable to other settings. Second, the time between the arrival of a patient suffering from cardiac arrest and the delivery of epinephrine in the ED might be a more appropriate indicator for determining whether the wearing of enhanced PPE has a negative effect on clinical tasks. However, precise timing data were lacking. Similarly, the number of shockable patients was inadequate to utilize the time interval between identifying a shockable rhythm and defibrillation as a proxy for the unfavorable effect of enhanced PPE. Therefore, in this study, the impact of enhanced PPE on CPR performance was evaluated in a limited manner. Third, the survey of this study included HCPs of different occupations, and their roles in the resuscitation procedures differed according to the occupational groups. Therefore, in the process of answering the questions regarding the detailed procedure, the respondents may have answered based on indirect experience of procedures that were not part of the respondent’s occupation. Fourth, we employed two different types of PAPR in this study, and we did not assess whether the outcomes would alter depending on the PAPR model. However, there were no significant differences in the shapes of the two devices, suggesting that the shape may have had little effect on the results. Fifth, although the ROSC and 1-month survival rates were lower in the enhanced PPE group than in the conventional PPE group (absolute differences of 5.5% and 4.7%, respectively), the difference was not statistically significant. The wide range of 95% CI in multivariable analysis suggests that this study may have limited power to detect statistically significant differences in outcomes between groups. Sixth, we used historical controls in our study. Due to the comparison of different study populations, the findings of our study may not be solely attributable to the level of PPE.

## 5. Conclusions

The use of enhanced PPE, including PAPR, affected the performance of CPR to some extent but did not alter patient outcomes. Although the HCPs were concerned about the consumption of resources and various inconveniences caused by wearing PAPR, they generally agreed that wearing enhanced PPE was necessary during the resuscitation of OHCA patients in the COVID-19 pandemic.

## Figures and Tables

**Table 1:** Baseline characteristics and outcomes of patients with the use of conventional and enhanced personal protective equipment.

Characteristics	Total *n* = 130	Conventional PPE *n* = 73	Enhanced PPE *n* = 57	*p*-Value
Age, years	68.0 (57.0–81.0)	68.0 (53.0–79.0)	66.0 (58.0–81.0)	0.840
Males	75 (57.6%)	41 (56.1%)	34 (59.6%)	0.690
Preexisting conditions				
Cardiovascular diseases	19 (14.6%)	11 (15.0%)	8 (14.0%)	0.717
Pulmonary diseases	21 (16.1%)	12 (16.4%)	9 (15.7%)	0.728
Chronic kidney disease	12 (9.2%)	9 (12.3%)	3 (5.2%)	0.248
Malignancy	26 (20.0%)	14 (19.1%)	12 (21.0%)	0.743
CVA	18 (13.8%)	15 (20.5%)	3 (5.2%)	0.022
Bystander witnessed	54 (41.5%)	31 (42.4%)	23 (40.3%)	0.808
Bystander CPR	80 (61.5%)	46 (63.0%)	34 (59.6%)	0.270
Pre-hospital CPR duration, min	23.0 (14.0–30.0)	24.0 (14.0–30.0)	22.0 (14.0–31.0))	0.852
Hospital CPR duration, min	16.0 (9.0–22.0)	15.0 (8.0–20.0)	18.0 (12.0–23.0)	0.030
Arrest location				
Public space	57 (43.8%)	33 (45.2%)	24 (42.1%)	0.724
Private space	73 (56.1%)	40 (54.7%)	33 (57.8%)	0.724
Initial non-shockable rhythm	123 (94.6%)	70 (95.8%)	53 (92.9%)	0.466
ETI attempt	115 (88.4%)	65 (89.0%)	50 (87.7%)	0.815
First success intubation	96 (83.4%)	51 (78.4%)	45 (90.0%)	0.130
First intubation trial interval, min	3.0 (2.0–4.0)	2.0 (1.0–3.0)	3.0 (2.0–5.0)	0.020
Interval to ABGA, min	6.0 (3.0–10.5)	3.0 (1.0–8.0)	8.0 (5.0–13.0)	<0.001
ROSC	61 (46.9%)	36 (49.3%)	25 (43.8%)	0.597
Alive ED discharge	29 (22.8%)	19 (26.3%)	10 (18.1%)	0.295
Targeted temperature management	11 (18.0%)	7 (19.4%)	4 (16.0%)	1.000
1-month survival	8 (6.1%)	6 (8.2%)	2 (3.5%)	0.465

All values are presented as medians (interquartile range) or numbers (percentages). PPE, personal protective equipment; CVA, cerebrovascular accident; CPR, cardiopulmonary resuscitation; ETI, endotracheal intubation; ABGA, arterial blood gas analysis; ROSC, return of spontaneous circulation; ED, emergency department.

**Table 2:** Association between clinical outcomes and enhanced personal protective equipment.

	Adjusted Odds Ratio	95% Confidence Interval	*p*-Value
ROSC			
Conventional PPE	ref		
Enhanced PPE	0.79	0.38–1.67	0.542
1-month survival			
Conventional PPE	ref		
Enhanced PPE	0.38	0.07–2.10	0.266

PPE, personal protective equipment; ROSC, return of spontaneous circulation.

**Table 3:** Comparison of healthcare professionals’ perceptions between the use of enhanced and conventional personal protective equipment.

Questions	Strongly Disagree *n* (%)	Disagree *n* (%)	Neither Agree Nor Disagree *n* (%)	Agree *n* (%)	Strongly Agree *n* (%)	Median (IQR)
Q1. Healthcare professionals’ performance on the following procedures was negatively affected by wearing enhanced PPE:						
Overall quality in CPR performance	1 (1.5)	8 (11.9)	11 (16.4)	38 (56.7)	9 (13.4)	4 (3–5)
Intubation	0 (0)	11 (16.4)	15 (22.4)	32 (47.8)	8 (11.9)	4 (3–4)
Intravenous line	3 (4.5)	10 (14.9)	18 (26.9)	30 (44.8)	6 (9.0)	4 (3–4)
ABGA	5 (7.5)	11 (16.4)	24 (35.8)	22 (32.8)	3 (4.5)	3 (3–4)
Chest compression	2 (3.0)	5 (7.5)	23 (34.3)	29 (43.3)	8 (11.9)	4 (3–4)
Medication administration	7 (10.4)	22 (32.8)	21 (31.3)	16 (23.9)	1 (1.5)	3 (2–4)
defibrillation	7 (10.4)	21 (31.3)	23 (34.3)	14 (20.9)	1 (1.5)	3 (2–3)
Patient monitoring	9 (13.4)	18 (26.7)	24 (35.8)	16 (23.9)	0 (0)	3 (2–3)
CPR instruction	2 (3.0)	3 (4.5)	10 (14.9)	42 (62.7)	9 (13.4)	4 (4–4)
Q2. It was easy to don and doff.	13 (19.4)	43 (64.2)	7 (10.4)	3 (4.5)	1 (1.5)	2 (2–2)
Q3. It was comfortable to breathe.	1 (1.5)	6 (9.0)	5 (7.5)	30 (44.8)	25 (37.3)	4 (4–5)
Q4. It suppressed heat buildup appropriately.	4 (6.0)	12 (17.9)	5 (7.5)	28 (41.8)	18 (26.9)	4 (3–5)
Q5. Contact with contaminants seemed to be reduced.	0 (0)	2 (3.0)	9 (13.4)	30 (44.8)	26 (38.8)	4 (4–5)
Q6. It was easy to secure a clear vision.	10 (14.9)	32 (47.8)	12 (17.9)	11 (16.4)	2 (3.0)	2 (2–3)
Q7. There were difficulties in communication between the medical staff.	3 (4.5)	1 (1.5)	0 (0)	27 (40.3)	36 (53.7)	5 (4–5)
Q8. The movement was limited.	1 (1.5)	5 (7.5)	8 (11.9)	39 (58.2)	14 (20.9)	4 (4–4)
Q9. It impeded each other’s movements among the medical staff.	1 (1.5)	6 (9.0)	8 (11.9)	33 (49.3)	19 (28.4)	4 (4–5)
Q10. There is a risk of contamination when doffing.	0 (0)	10 (14.9)	21 (31.3)	28 (41.8)	8 (11.9)	4 (3–4)

PPE, personal protective equipment; CPR, cardiopulmonary resuscitation; SD, standard deviation; ABGA, arterial blood gas analysis.

## Supplementary Materials

The following are available online at https://www.mdpi.com/article/10.3390/medicina57121291/s1, Table S1: Baseline characteristics of the survey respondents, Table S2: Survey questions and answers on wearing powered air purifying respirator during resuscitation of out-of-hospital cardiac arrest patients, Table S3: Survey questions and answers regarding the necessity of wearing powered air purifying respirator and their reasons, Table S4: Free opinions on the use of enhanced personal protective equipment (advantages, disadvantages, and improvement plan in use)
