# Supplementary material for: Impact of Personal Protective Equipment on Out-of-Hospital Cardiac Arrest Resuscitation in Coronavirus Pandemic
Source: Medicina (Kaunas). 2021 Nov 24;57(12):1291. doi: 10.3390/medicina57121291 (PMC8708039; doi:10.3390/medicina57121291)
Supplement: Supplementary file 1 [file medicina-57-01291-s001.zip › medicina-1463347-supplementary.pdf]

Supplementary Material

Table S1. Baseline characteristics of the survey respondents

	MD N=27	RN+EMT N=40	Total N=67
**Age in years, mean (SD)**	34.2 (6.9)	30.0 (4.3)	31.7 (5.8)
**Males, N (%)**	14 (51.8%)	8 (20.0%)	22 (32.8%)
**Experience as emergency department worker, N (%)**			
≤2 years	8 (29.6%)	6 (15.0%)	14 (20.9%)
>2 and ≤5 years	8 (29.6%)	14 (35.0%)	22 (32.8%)
>5 and ≤10 years	3 (11.1%)	13 (32.5%)	16 (23.9%)
>10 years	8 (29.6%)	7 (17.5%)	15 (22.4%)
**Type of CPR certification, N (%)**			
BLS provider	12 (44.4%)	27 (67.5%)	39 (58.2%)
BLS instructor	4 (14.8%)	2 (5.0%)	6 (9.0%)
ACLS provider	19 (70.4%)	26 (65.0%)	45 (67.2%)
ACLS instructor	7 (25.9%)	3 (7.5%)	10 (14.9%)
PALS provider	1 (3.7%)	5 (12.5%)	6 (9.0%)
**Number of CPR performances while wearing PAPR, N (%)**			
<10 times	13 (48.5%)	23 (57.5%)	36 (53.7%)
10–19 times	9 (33.3%)	14 (35.0%)	23 (34.3%)
≥20 times	5 (18.5%)	3 (7.5%)	8 (11.9%)

MD, medical doctor; RN, registered nurse; EMT, emergency medical technician; SD, standard deviation; CPR, cardiopulmonary resuscitation; BLS, basic life support; ACLS, advanced cardiovascular life support; PALS, pediatric advanced life support; PAPR, powered air purifying respiratory

Table S2. Survey questions and answers on wearing powered air purifying respirator during resuscitation of out-of-hospital cardiac arrest patients

Questions	Survey scores, Mean (SD)			
	Total (N=67)	MD (N=27)	RN+EMT (N=40)	P-value
**Q1. Health care workers' performance on the following procedures was negatively affected by wearing enhanced PPE.**				
**1.** Overall quality in CPR performance	3.7 (0.9)	4.0 (0.9)	3.5 (0.9)	0.039
**2.** Intubation	3.6 (0.9)	3.8 (0.8)	3.4 (0.9)	0.108
**3.** Intravenous line	3.4 (1.0)	3.6 (0.8)	3.3 (1.1)	0.262
**4.** ABGA	3.1 (1.0)	3.3 (0.8)	3.0 (1.1)	0.203
**5.** Chest compression	3.5 (0.9)	3.6 (0.9)	3.5 (0.9)	0.686
**6.** Medication administration	2.7 (1.0)	3.1 (0.8)	2.5 (1.0)	0.009
**7.** Defibrillation	2.7 (1.0)	3.0 (0.9)	2.5 (1.0)	0.081
8. Patient monitoring	2.7 (1.0)	3.0 (1.0)	2.5 (1.0)	0.074
9. Resuscitation instruction	3.8 (0.8)	3.9 (0.9)	3.8 (0.8)	0.699
**Q2. It was easy to don and doff.**	2.0 (0.8)	2.0 (0.9)	2.1 (0.7)	0.705
**Q3. It was comfortable to breathe.**	4.1 (1.0)	4.1 (1.2)	4.1 (0.8)	0.803
**Q4. It suppressed heat buildup appropriately.**	3.7 (1.2)	4.0 (1.1)	3.5 (1.3)	0.093
**Q5. Contact with contaminants seemed to be reduced.**	4.2 (0.8)	4.3 (0.7)	4.1 (0.8)	0.234
**Q6. It was easy to secure a clear vision.**	2.4 (1.0)	2.6 (1.1)	2.3 (1.0)	0.239
**Q7. There were difficulties in communication between the medical staff.**	4.4 (0.9)	4.4 (1.1)	4.4 (0.8)	0.584
**Q8. The movement was limited.**	3.9 (0.9)	3.7 (1.0)	4.0 (0.7)	0.140
**Q9. It impeded each other's movements among the medical staff.**	3.9 (1.0)	3.7 (1.1)	4.1 (0.8)	0.160
**Q10. There is a risk of contamination when doffing.**	3.5 (0.9)	3.4 (0.9)	3.6 (0.9)	0.639

MD, medical doctor; RN, registered nurse; EMT, emergency medical technician; SD, standard deviation; CPR, cardiopulmonary resuscitation; ABGA, arterial blood gas analysis; PAPR, powered air purifying respirator

Table S3. Survey questions and answers regarding the necessity of wearing powered air purifying respirator and their reasons

	N	%
**Do you think enhanced PPE is necessary during the resuscitation of OHCA patients? (Yes)**	61	91.0
**Why do you think PAPR is necessary during the resuscitation of OHCA patients?**		
A. We can participate in patient management feeling self-protected	27	44.3
B. It has a positive impact on the reduction of infection rates	24	39.3
C. We need overprotection in this situation though I am not sure whether it will help to reduce the infection rate	15	24.6
**Why do you think PAPR is unnecessary in the resuscitation process of OHCA patients?**	5	7.5
A. Conventional PPE is sufficient	4	80.0
B. I think the probability of being infected with COVID-19 from patients with OHCA is very low	1	20.0
C. Too many resources required such as cost/equipment management	3	60.0
D. Disadvantages in medical treatment caused by cumbersome PPE outweigh the advantages	3	60.0

PAPR, powered air purifying respirator; PPE, personal protective equipment; OHCA, out-of-hospital cardiac arrest; COVID-19, coronavirus disease

Table S4. Free opinions on the use of enhanced personal protective equipment (advantages, disadvantages, and improvement plan in use)

	N
**Negative opinions**	
Difficulty in communication due to the noise of the device	24
Self-contamination risk when doffing	10
Battery life issue	9
Difficulty in auscultation	8
The long donning and doffing times cause a delay in starting CPR	7
Relative CPR manpower shortage due to the lack of PAPR quantity	7
Discomfort when donning and doffing	4
Concerns about fan malfunction and machine failure	4
Necessity of equipment inspection and re-setting before and after CPR	3
Problems with medical treatment due to limited movement	3
Assistance and monitoring are required when donning and doffing	2
Difficulty in securing visibility	2
Dull feeling when moving, interruption between medical personnel while moving	2
Dangerous for those who wear glasses because the glasses come off when doffing	2
N95 masks slipped off when doffing the hood	2
Simulation training is required regularly to prevent CPR quality loss	2
Concerns about equipment obsolescence due to equipment reuse	2
Poor durability of PAPR hood	1
PAPR hood keeps coming off	1
Headache caused by machine noise	1
PAPR hood size adjustment is difficult	1
Protection is questionable because the fitting test is not performed	1
Inconvenient during patient history taking and recording EMR	1
**Positive opinions**	
Wearing PAPR helps to prevent infection	2
Temperature control is easy, so we can work comfortably in CPR situations	1
It was easy to breathe while wearing PAPR hood	1

PAPR, powered air purifying respirator; CPR, cardiopulmonary resuscitation; EMR; Electronic medical record
